# Cylindrospermopsin toxicokinetics: in silico ADMET modeling, rat and human liver microsome metabolism, and UHPLC-MS/MS metabolite characterization

**DOI:** 10.1007/s00204-026-04404-0

**Published:** 2026-04-28

**Authors:** Antonio Cascajosa-Lira, Remedios Guzmán-Guillén, Ana M. Cameán, Angeles Jos, Ana Isabel Prieto

**Affiliations:** Area of Toxicology, Faculty of Pharmacy, University of Sevilla, Seville, Spain

**Keywords:** Cylindrospermopsin, In silico, In vitro, Microsomes, Metabolites, UHPLC-MS/MS

## Abstract

Cylindrospermopsin is a potent cyanobacterial toxin of growing global concern due to its environmental persistence and broad toxicological profile. Despite increasing evidence of its toxic effects, knowledge of its toxicokinetics remains limited. This study integrates in silico ADMET predictions with in vitro microsomal assays using human and sex-stratified rat liver microsomes to characterize CYN biotransformation. In silico predictions indicated low intestinal absorption, improbable blood–brain barrier (BBB) penetration, minimal binding to plasma proteins, and very limited CYP450-mediated metabolism. However, several potential phase II conjugation routes were identified, with certain metabolites predicted to display altered toxicological properties compared with the parent compound. Complementary in vitro studies in microsomes confirmed the high metabolic stability of CYN, with only modest decreases in toxin concentration observed during incubation (8–15% decrease). Notably, biotransformation was more evident under phase II conditions, particularly glucuronidation and amino acid conjugation. Novel metabolites were detected for the first time by Ultra-High Performance Liquid Chromatography (UHPLC-/MS/MS), including conjugates with cysteine, glycine, taurine, and arginine, as well as with fatty acids. No significant quantitative differences were observed between sexes or species, although qualitative variability in metabolite profiles was detected. These findings provide new insights into the metabolic fate of CYN, underscore the importance of conjugation reactions in its detoxification, and support the application of integrative computational and experimental approaches to improve cyanotoxin risk assessment.

## Highlights

- First integrative in silico ADMET and in vitro microsomal metabolism study of CYN.
- In silico predictions showed minimal absorption, BBB crossing, and CYP450 activity.
- In vitro study showed CYN’s high stability, with phase II over phase I metabolism.
- First identification of novel metabolites: conjugates with amino acids and lipids.
- No major species/sex differences, although variability in metabolites was observed.

## Introduction

Cylindrospermopsin (CYN) is a potent cyanobacterial toxin with increasing global relevance due to its detection in freshwater bodies across tropical and temperate regions, driven by eutrophication and climate change (Zhang et al. 2025). Since that discovery, CYN-producing species—including *Raphidiopsis raciborskii, Chrysosporum ovalisporum, Raphidiopsis curvata,* and *Umezakia natans*, among others—have been isolated in diverse regions worldwide (Adamczuk et al. 2025; Harada et al. 1994; Li et al. 2001; Schembri et al. 2001). The toxin’s tricyclic guanidinium–sulfate–uracil core makes it highly water-soluble, and research has demonstrated that it remains stable in the environment, with minimal breakdown through hydrolysis, light-induced degradation, or microbial activity under common temperature and pH conditions, properties that enable its accumulation in drinking and recreational water sources, plants and different species of animals (De La Cruz et al. 2013; Pichardo et al. 2017; Zhang et al. 2025).

Toxicologically, CYN is classified as a multi-organ protein synthesis inhibitor exhibiting hepatotoxic, nephrotoxic, neurotoxic, endocrine, genotoxic, immunotoxic, and possibly carcinogenic, and reproductive toxic effects (Casas-Rodríguez et al. 2025a; Chernoff et al. 2011; Díez-Quijada et al. 2019; Guzmán-Guillén et al. 2015; Yang et al. 2021; Zhang et al. 2025). Research indicates that CYN is likely to disrupt protein synthesis and deplete intracellular glutathione (GSH) levels (Froscio et al. 2003; Runnegar et al. 1994, 1995) and potentially interacting with cytochrome P450 (CYP450) (Runnegar et al. 1995; Yang et al. 2021). In comparison to toxicity evaluation studies, data on CYN toxicokinetics (ADME: absorption, distribution, metabolism and excretion) are more limited (WHO, 2020). Metabolism is a key determinant in modulating the toxicity of many chemicals, as original compounds can be transformed into highly toxic metabolites (Cascajosa-Lira et al. 2023). Studies indicate that CYP450 enzymes may be involved in the metabolic activation of CYN, with CYP450 inhibition reducing toxicity and covalent binding of metabolites to DNA; this suggests that bioactivation may occur (Humpage et al. 2005; Norris et al. 2002; Runnegar et al. 1995; Shaw et al. 2000). To date, only one work focused on oxidative stress in brain extracts of rats orally exposed to CYN identified phase I and phase II metabolites of this toxin (Plata-Calzado et al. 2025).

Runnegar et al. (1995) suggested that CYN-induced GSH depletion results from impaired synthesis, while Norris et al. (2002) argued that such depletion, whether by conjugation or inhibition of synthesis, is not central to CYN hepatotoxicity. In contrast, our group observed increased γ-glutamylcysteine synthetase (γ-GCS) activity and a low GSH/GSSG ratio in tilapia exposed to CYN, indicating that GSH synthesis is not impaired (Guzmán-Guillén et al. 2013).

More recently, Adamski and Kaminski (2021) conducted an in vitro study to evaluate the changes of the antioxidant properties of GSH in presence of CYN and its decomposition products (at 1, 5 and 10 µg/mL), and their results indicated no significant interaction between CYN and GSH under in vitro conditions. However, the experiment focused solely on the direct chemical interaction between GSH and CYN or its degradation products, without the involvement of enzymes or other intracellular compounds that could influence CYN reactivity. Therefore, the exact mechanism underlying the interaction between CYN and GSH remains unclear.

Norris et al. (2001) identified the urinary system as the primary excretion pathway for CYN, with only a small portion potentially bound to proteins. This suggests that some CYN is excreted unmetabolized and may be detectable in its original form in urine.

Understanding ADME is essential to interpret dose-behavior relationships, exposure models, and risk assessment. Due to experimental challenges and limited animal-to-human extrapolation, combining in silico predictions with empirical models can streamline research efforts in its early stages. Computational ADMET tools can forecast absorption, distribution, P450-mediated metabolism, excretion, and toxicity predictions for CYN and possible metabolites. These in silico approaches provide valuable insights into the molecular-level interactions between bioactive compounds and their enzymatic or organ targets (Brogi et al. 2020). In the case of CYN, previous studies from our laboratory demonstrated potential interactions between CYN and androgen receptor (Chernoff et al. 2011). In addition, in silico methods help prioritize targets for in vitro validation and highlight high-risk metabolic products, guiding experimental strategies.

Following computational insights, in vitro studies with pooled human and sex-stratified rat liver microsomes can elucidate metabolic rates, enzyme kinetics, metabolite profiles, and identify potential bioactivated intermediates. Microsomes can be valuable tools for identifying the specific microsomal enzymes responsible for the metabolism of a compound, which is important for extrapolating findings across different species. In addition, co-analysis with high-resolution mass spectrometry can detect and characterize phase I/II metabolites, including glutathione conjugates, hydroxylated or desulphonated species (Cascajosa-Lira et al. 2023; Chavan et al. 2018; Feng et al. 2021; Johnsi Rani et al. 2018; Lee et al. 2015; Plata-Calzado et al. 2025). Research has consistently demonstrated significant sex-based differences in microsomal CYP450 activity, particularly in rats, which may have important implications for both toxicity and the extrapolation of metabolic data to humans. For instance, rats exhibit pronounced sex-specific expression of CYP isoforms, such as CYP2C and CYP3A families, with males and females showing distinctly different enzymatic profiles (Mugford and Kedderis 1998).

Concerning other cyanotoxins, such as microcystins (MCs), studies of their conjugation with GSH have been performed (Buratti et al. 2013; Gehringer et al. 2004). In the case of CYN, the only study performed to date using HepaRG cells, Human Liver Microsomes (HLM) and rat and human S9 liver tissue fractions found CYN recovery around 100% in these systems at the end of the incubation period, and no detectable phase I metabolites of the toxin were reported (Kittler et al. 2016). Therefore, more comprehensive data on the in vitro metabolism of CYN across species is needed to enhance risk assessment efforts.

Considering this background, and according to the next generation risk assessment principles, the objective of the present work is to perform for the first time a combined in silico ADMET modeling and in vitro microsomal metabolism studies for CYN. This study incorporates male and female rat, and human microsomes, to identify potential metabolic differences associated with species or sex. The present research would help to understand CYN metabolism and to identify potential metabolites. The identification of CYN metabolites was performed by UHPLC-/MS/MS.

## Methods

### Chemicals and reagents

Cylindrospermopsin (95% purity) was supplied by Enzo Life Sciences (Switzerland). PBS, testosterone, 7-hydroxycoumarin, reduced glutathione, Glucose-6-phosphate dehydrogenase (G6PDH), magnesium chloride (MgCl_2_), tris buffer and uridine 5′-diphosphoglucuronic acid (UDPGA) were purchased from Sigma-Aldrich (St. Louis, MO, USA). HPLC-grade methanol (MeOH) and acetonitrile (ACN) were obtained from Merck (Darmstadt, Germany). Pooled Rat Liver Microsomes (RLM) of both sexes and HLM, were supplied by Corning Gentest (Woburn, Massachusetts, USA) and Gibco (Biomol, Sevilla, Spain). Alamethicin, NADP^+^ and Glucose-6-phosphate (G6P) were purchased from Cayman Chemical Company (Ann Arbor, Michigan, USA). Ultrapure water (18.2 MΩ cm resistivity) purified by the NANOpure Diamond™ (Barnstead, USA) system was employed.

### In silico approach

The chemical structures of CYN metabolites resulting from different biotransformation routes were obtained and converted to SMILES with the software Biotransformer 3.0. Subsequently, the SMILES were submitted to ADMETlab 3.0 software to predict ADME parameters and toxic effects of CYN and its identified metabolites. Regarding absorption, Caco-2 and MDCK permeability, as well as human intestinal absorption (HIA) were predicted. In the distribution, the probability of plasma protein binding (PPB) and crossing blood–brain barrier (BBB) were calculated. Concerning metabolism, the probability of acting as inhibitor or substrate of different CYP isoforms were considered. As excretion parameters, plasma clearance (CL_plasma_) and half-life (T_1/2_) were calculated. To assess potential toxicity of CYN and its metabolites, the following endpoints were predicted: Ames test, carcinogenicity, human hepatotoxicity, genotoxicity, drug-induced nephro- and neurotoxicity.

### In vitro metabolism

The assays were conducted following the methodology described in Lee et al. (2015) and Cascajosa-Lira et al. (2023), with minor modifications based on Feng et al. (2021) and Johnsi Rani et al. (2018). Three experimental groups were established: one representing phase I (oxidation) and two representing phase II (conjugation with GSH or glucuronic acid). Separate reactions were assessed with microsomes from male (MRLM) or female (FRLM) rats, as well as HLM.

The final volume in the metabolic reaction mixtures was 1 mL. It consisted of: *a)* MRLM/FRLM/HLM (0.5 mg/mL) in phosphate buffer saline (PBS) (100 mM, pH 7.4) – in the case of GSH conjugation, GSH (5 mM) was also added—and this mixture was incubated at 37 °C (5 min, 50 r.p.m.); *b)* CYN (2 ppm, 5 µM) or positive controls for oxidation and glucuronidation reactions (20 µM testosterone, and 1 µM 7-hydroxycoumarin, respectively); a positive control is not required for GSH conjugation, as the reaction can occur both in the presence and absence of glutathione S-transferase; *c)* NADPH regenerating system for oxidation and GSH conjugation reactions, or UGT reaction mix for glucuronidation, to start the reactions. NADPH regenerating system includes G6PDH (0.4 U/mL), MgCl_2_ (3.3 mM), NADP^+^ (1.3 mM), and G6P (3.3 mM). The UGT reaction mix is composed of Tris–HCl (50 mM, pH 7.5), MgCl_2_ (8 mM), alamethicin (25 µg/mL, dissolved in 1.25% MeOH), and UDPGA (2 mM). Positive controls, testosterone and 7-hydroxycoumarin, were employed to confirm the enzymatic activity of CYP450 and glucuronosyltransferase, respectively. A blank without toxin was also included in each reaction, and all reactions were performed in triplicate.

Once all reagents have been added, all samples were incubated at the conditions previously described, and aliquots of 100 µL were taken at different times (0, 1, 3, 5, 15, 30, 60 and 120 min) and mixed with the same volume of cold ACN, to stop the metabolic reactions. Immediately, samples were vortexed and kept frozen until analysis. The selected incubation time was established in accordance with the stability limits of the microsomal preparations, as specified by the manufacturer’s instructions. CYN concentration (5 µM) was selected based on two criteria: first, it is within the same molar range as the positive controls, ensuring adequate enzymatic availability and comparability across reactions; second, it represents an environmentally relevant concentration, as similar levels have been reported in certain cyanobacterial bloom events (Yang et al. 2021).

### UHPLC-MS/MS: chromatographic conditions and data acquisition

Chromatographic analysis was performed using a Thermo Scientific Liquid Chromatography system consisting of a binary UHPLC Dionex Ultimate 3000 RS connected to a quadrupole-orbitrap Qexactive hybrid mass spectrometer (ThermoFisher Scientific, USA) equipped with a Waters C18 Acquity HSST3 column (10 × 2.1 mm, 1.8 μm) maintained at 35 °C, with a HESI ion source operated in positive mode (spray voltage: 3,500 V; capillary temperature: 320 °C; sheath gas: 50 units; auxiliary gas: 12.5 units; probe heater temperature: 425 °C; S-Lens RF level: 50). The autosampler was kept at 15 °C. Mobile phase A consisted of water with 0.1% formic acid and mobile phase B of ACN with 0.1% formic acid, delivered at a flow rate of 0.45 mL/min under the following gradient: 2% B from 0.0 to 7.0 min, ramped to 70% B at 7.0 min, to 100% B at 7.1 min, held until 8.0 min, and then returned to 2% B by 8.1 min until the end of the run at 10.0 min. The mass spectrometer was operated with a Data Dependent Analysis in a full scan range of *m/z* 100–1500 at a resolution of 70,000 (FWHM), AGC target of 3 × 10^6^, and a maximum injection time of 200 ms, with the top 5 most intense ions (Top5) subjected to subsequent MS/MS fragmentation for further analysis. The extracted ion chromatograms were generated using TraceFinder 5.1 software to specifically search for the exact masses of CYN (*m/z* 416.12345), testosterone (*m/z* 289.21621), and 7-hydroxycoumarin (*m/z* 163.03897) within mass error of 5 ppm.

### Metabolites characterization: data analysis

For comprehensive metabolite profiling (phases I and II), data were further analyzed using Compound Discoverer™ 3.2 (Thermo Fisher Scientific, Waltham, MA, USA) following the workflow: spectra selection, retention time alignment, and compound identification based on accurate mass and MS/MS fragmentation patterns, either by comparison with authentic standards or through searches in various databases (MassBank, mzCloud, ChemSpider, and an internal laboratory database of mass spectrometry service of University of Seville).

### Statistical analysis

Results are presented as mean ± standard deviation (SD). Statistical evaluations were conducted using GraphPad Prism 9 (GraphPad Software Inc., La Jolla, CA, USA). Data normality was assessed with the Kolmogorov–Smirnov test prior to analysis. A one-way ANOVA was applied for normally distributed data, followed by the Tukey–Kramer post hoc test when significant differences were detected. For datasets that did not meet normality criteria, the Kruskal–Wallis test was employed, with Dunn’s multiple comparison test for pairwise analysis. A p-value below 0.05 was considered indicative of statistical significance.

## Results and discussion

### In silico outcomes

**Fig. 1:**
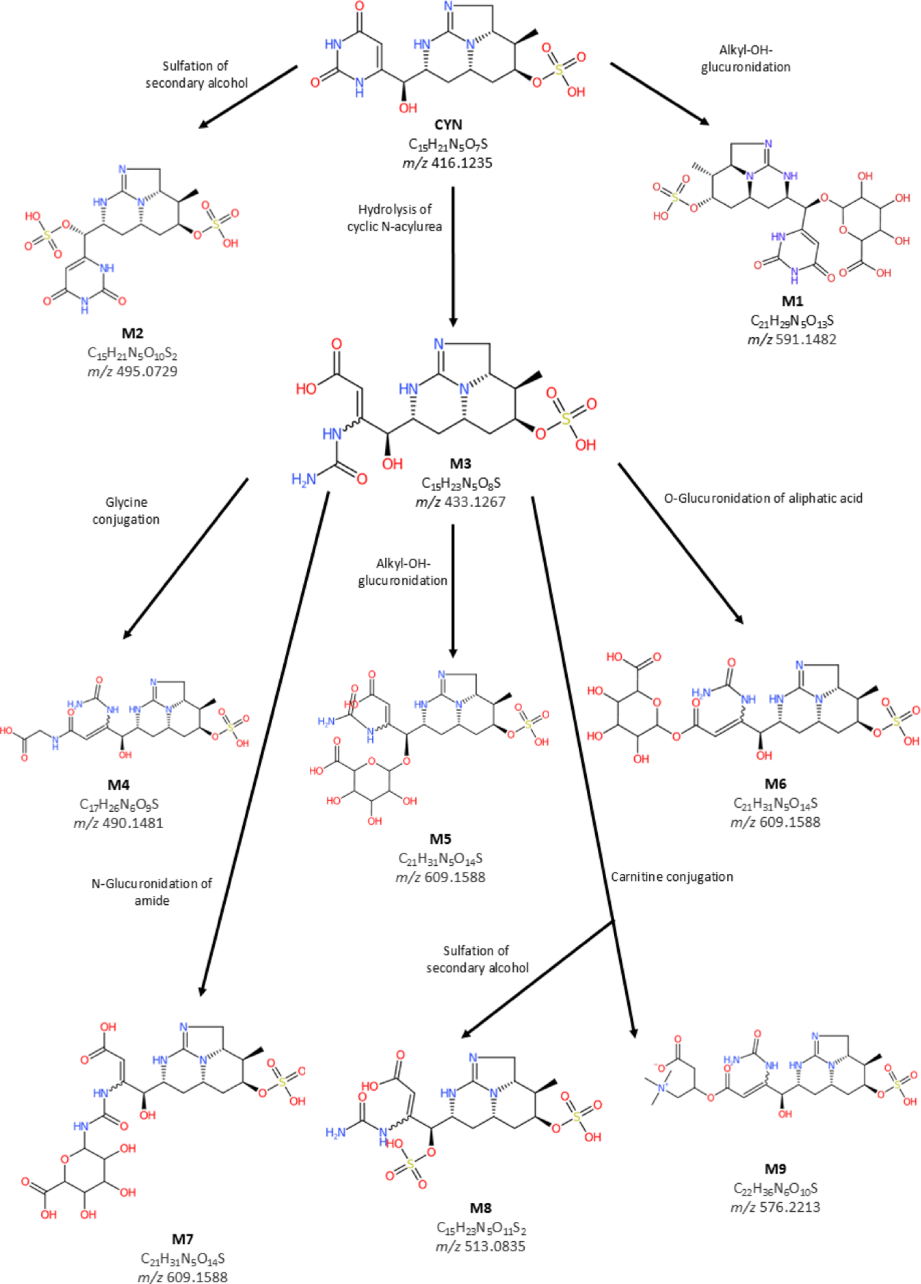
Predicted metabolic pathway of CYN according to the results obtained from the in silico metabolism assay performed with Biotransformer 3.0 software

The predicted metabolites (M1-M9) obtained with Biotransformer 3.0 software are shown in Fig. 1. The primary pathway of CYN metabolism is alkyl-OH-glucuronidation (M1), sulfation (M2), and hydrolysis of cyclic N-acylurea (M3). Secondary metabolism appears to affect only to M3: three different glucuronidations, conjugation with glycine and carnitine, and sulfation.

**Fig. 2:**
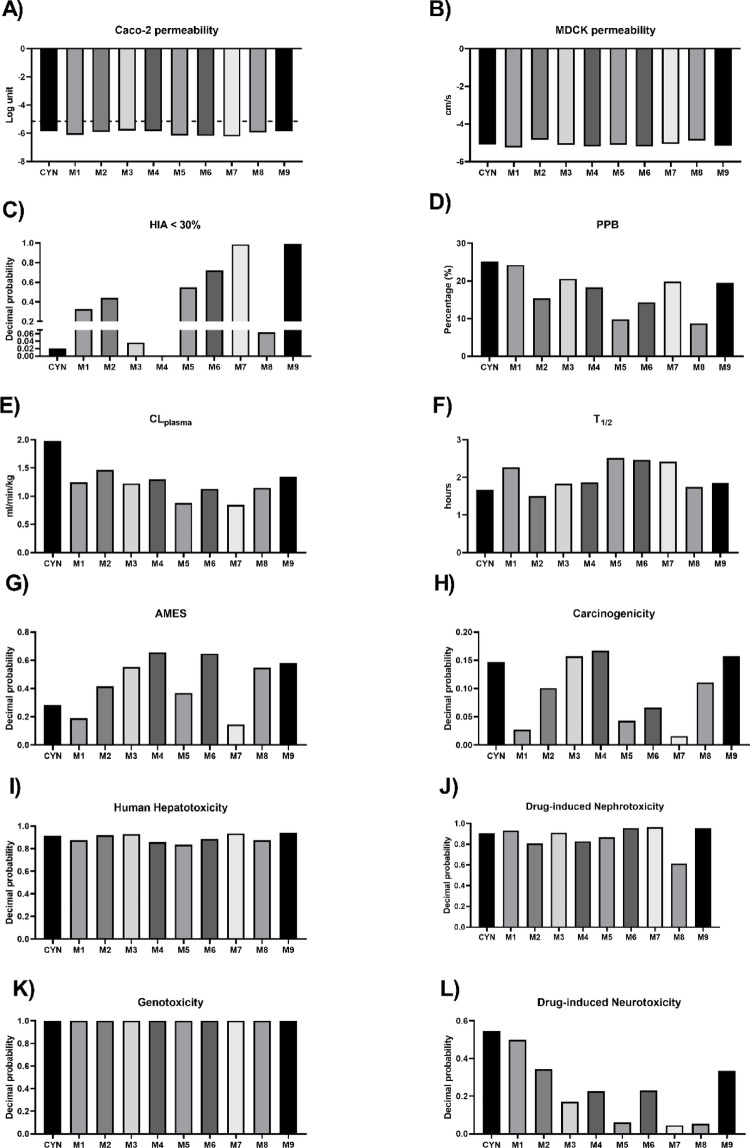
Predictions of different kinetic and toxicological parameters of CYN and its metabolites (M1-M9). **A** Caco-2 permeability (Log units); optimal: higher than − 5.15 Log unit. **B** MDCK permeability (cm/s); low permeability: < 2 × 10^−6^ cm/s; medium permeability: 2–20 × 10^−6^ cm/s; high passive permeability: > 20 × 10^−6^ cm/s. **C** Human Intestinal Absorption, within the range of 0 to 1, showing the probability of being absorbed; **D** Plasma Protein Binding (%); optimal: < 90%; **E** Plasma clearance (CL_plasma_; ml/min/kg); high clearance: > 15 ml/min/kg; moderate clearance: 5–15 ml/min/kg; low clearance: < 5 ml/min/kg; **F** Half-life (T_1/2_, hours); ultra-short half-life: < 1 h; short half-life: 1–4 h; intermediate short half-life: 4–8 h; long half-life: > 8 h; **G** AMES test, within the range of 0 to 1, where 0: AMES ( −), and 1: AMES ( +); **H** Carcinogenicity, within the range of 0 to 1, where 0: non-carcinogens, and 1: carcinogens; **I** Human Hepatotoxicity, within the range of 0 to 1, where 0: non-hepatotoxic, and 1: hepatotoxic; **J** Drug-induced Nephrotoxicity, within the range of 0 to 1, where 0: non-nephrotoxic, and 1: nephrotoxic; **K** Genotoxicity, within the range of 0 to 1, where 0: non-genotoxic, and 1: genotoxic; **L** Drug-induced Neurotoxicity, within the range of 0 to 1, where 0: non-neurotoxic, and 1: neurotoxic

Figure 2 reflects the main kinetic and toxicological parameters of CYN and its metabolites (M1-M9) predicted by ADMETlab 3.0 software. The observed parameters regarding the uptake (Caco-2 and MDCK permeability, and HIA) (Fig. 2A-C) show a restricted probability of absorption for CYN (2% of HIA), in agreement with the results in vitro (Fernández et al. 2014; Pichardo et al. 2017). Also, Plata-Calzado et al. (2024) observed a limited uptake of CYN on THP-1 (monocytes) cultures with a concentration-dependent pattern. Thus, at the highest exposure concentration (3 µg/mL), 1.30% and 65% CYN was detected at intracellular and extracellular level, respectively. In the case of metabolites, the absorption probability continues to be low for Caco-2 and renal cells, but regarding HIA, results vary depending on the compound: it is very low in the case of M3 (3.6%) and especially M4 (0.1%), but it is increased for M7 (98.6%) and M9 (98.9%). However, there are no in vivo studies about the intestinal absorption of CYN and its potential metabolites. A recent study from our group conducted an oral exposure of CYN in rats (500 µg/kg b.w.), and identified the toxin in the intestines by Matrix-assisted laser desorption/ionization mass spectrometry imaging (MALDI-MSI) and its sodium and potassium adducts in the chyme mixture up to approximately 2 h post-exposure, with a progressive decrease along the intestinal tract, but without evidence of epithelial uptake or systemic entry Casas-Rodríguez et al. (2025b). The same study reported alterations in lipid families associated with the inflammatory response, increased oxidative stress, and progressive disruption of cell membrane integrity. These findings strongly suggest that CYN, or possibly one or more of its metabolites, may exert local toxic effects on the intestinal epithelium. In spite of the reports and results suggesting limited uptake of CYN and/or its metabolites, there is an important weight of evidence regarding its toxicity. Therefore, it is important to evaluate its potential risks for human health. In this sense, ADMET and in vitro metabolism results provide new insight into which metabolites may potentially permeate via the intestinal barrier, supporting hypotheses for in vivo uptake that warrant follow-up studies.

The parameters evaluating the distribution of CYN within the organism indicate that both CYN and its potential metabolites exhibit a low probability of binding to plasma proteins (Fig. 2D) and a negligible likelihood of crossing the BBB (0%). However, our *in silico* studies are limited, as they cannot predict the organotropism of a substance. In vivo studies have demonstrated that CYN can be distributed mainly in the intestine, liver, and kidney following exposure in fish (Gutierrez-Praena et al., 2014; Guzmán-Guillén et al. 2014). To date, the detection of CYN in the brains of fish exposed to the toxin has only been reported in two studies conducted using ELISA tests (Da Silva et al. 2018; Guzmán-Guillén et al. 2015), which provides evidence of its ability to cross the BBB. However, the possibility of false positives for CYN determination in the ELISA test has raised some concerns. Contrary to the in silico prediction, in vivo, Plata-Calzado et al. (2025) detected 14 potential CYN-derivate compounds in brain samples of rats exposed orally to CYN as well as changes in different parameters (acetylcholinesterase activity and oxidative stress biomarkers). Therefore, the interaction of CYN with the BBB is an important issue that warrants further investigation.

The probability of CYN and its metabolites of being metabolized by the different cytochrome isoforms (CYP1A2, CYP2C19, CYP2C9; CYP2D6, CYP3A4, CYP2B6, CYP2C8) is very low; for this reason, it has not been represented in any figure, as the vast majority of the predicted values are zero, with only one exception: CYP2C9 for M2, M4, M5, M6, M7, and M8. Similarly, the probability of interaction with HLM is very low for both, CYN and its metabolites (data not shown).

Regarding the excretion processes of CYN and its metabolites, both Cl and T_1/2_ were calculated (Fig. 2E, F). In all cases, Cl values were below 5 mL/min/kg, indicating low clearance. However, the T_1/2_ values ranged between 1 and 2.5 h, reflecting a short half-life. Although a low plasma clearance (Cl) would typically be associated with a long elimination half-life, this expectation changes when the volume of distribution (Vd) is extremely low (Mansoor and Mahabadi 2023). For both CYN and its predicted metabolites, the reduced Vd indicates that these compounds remain mostly confined to the plasma rather than being widely distributed in body tissues. This is consistent with the well-known hydrophilic characteristic of the toxin [16]. Moreover, it is worth noting that the health-based guidance values established by WHO for CYN was based on kidney damage. Under these conditions, even with low clearance, the total amount of substance present in the body is limited, which can result in faster elimination and a shorter half-life. The Vd was also calculated for CYN and its predicted metabolites; however, due to the extremely low values obtained, it was not represented in the figure. Previous studies have attempted to characterize the kinetic elimination processes of CYN; however, their interpretation has been limited by the considerable variability reported in fecal excretion, ranging from 0.7 to 55.8%. In addition, these studies relied on the use of radiolabeled toxin without calculating the corresponding pharmacokinetic parameters (clearance and half-life) (Norris et al. 2001).

The in silico results show probability for mutagenicity for CYN and its metabolites (Fig. 2G), previously demonstrated in vitro (Puerto et al. 2018). This finding contrasts with the predicted carcinogenicity (Fig. 2H), which suggested that CYN metabolites are less toxic than the parent compound. In terms of other toxicological endpoints, the predicted potential of CYN and its metabolites to induce hepatotoxicity, nephrotoxicity, and genotoxicity (Fig. 2I-K) remained similar, indicating that bioactivation is not necessary for these effects to occur. Conversely, the neurotoxic potential (Fig. 2L) was reduced in metabolites compared with CYN, suggesting a detoxification process in relation to neurotoxicity. It has been established that the uracil ring structure of CYN is essential for its toxic activity (Banker et al. 2001). In our study, those metabolites that retained the uracil ring intact or exhibited only slight modifications (M3, M5, M6) appeared to be associated with increased mutagenicity activity as reflected in the Ames test or the preservation of carcinogenicity (only in the case of M3). In contrast, metabolites undergoing significant structural alterations of the uracil ring showed a marked reduction in toxicity in most of the parameters evaluated. It is well established that CYN can exert toxic effects in various organs (Chernoff et al. 2018); however, it remains unclear whether prior bioactivation is required for these effects to occur. While some genotoxicity and mutagenicity studies suggest that bioactivation is necessary, definitive evidence is still lacking (Puerto et al. 2018).

### In vitro metabolism

**Fig. 3:**
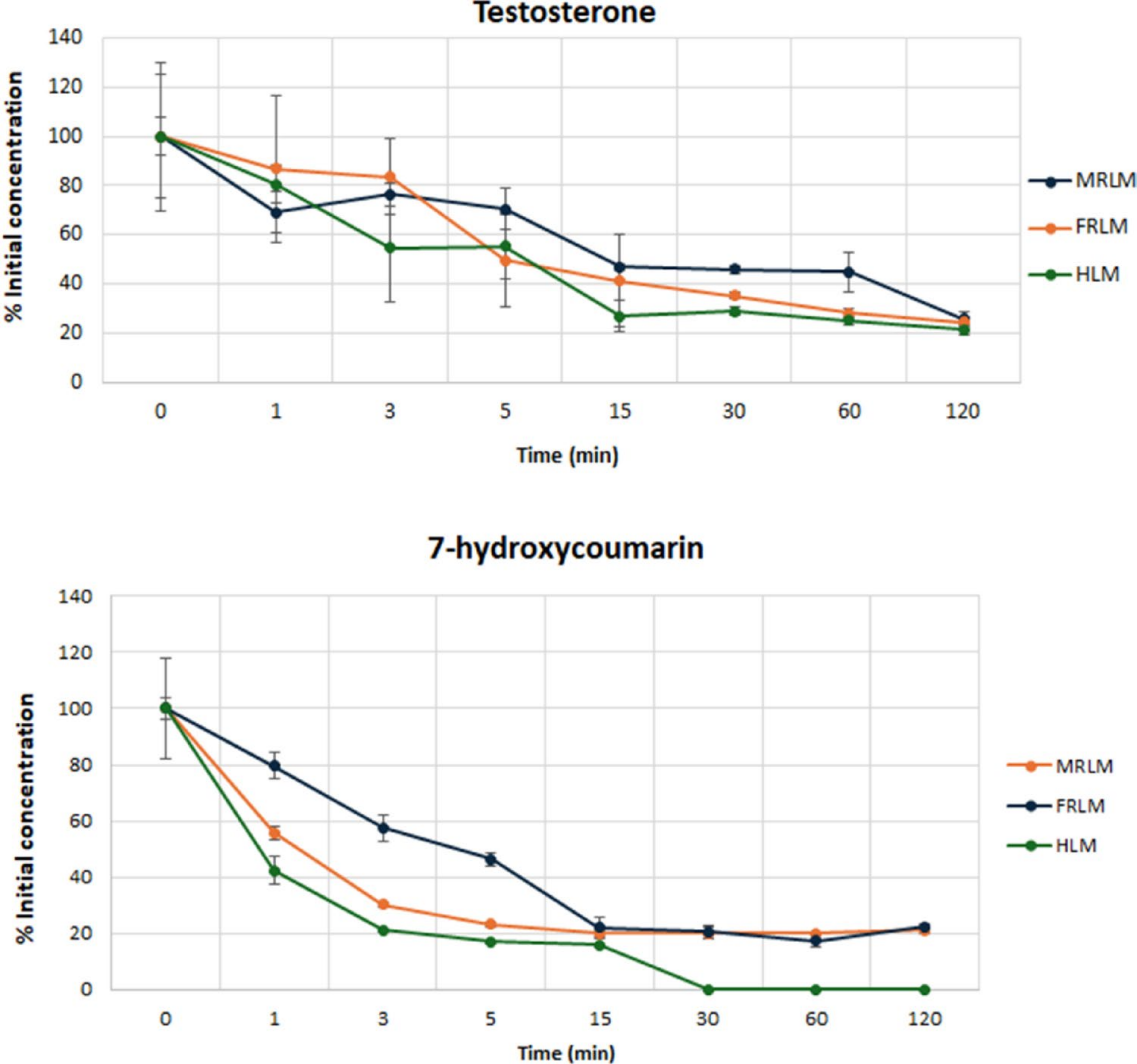
Metabolic stability of testosterone and 7-hydroxycoumarin in pooled rat (male and female) and human liver microsomes. Data are expressed as the means ± SD of triplicate runs

After the incubation period with the three different microsomes (male rat, female rat, and human), the positive controls (Fig. 3) confirmed the optimal metabolic activity of the enzymes present in the metabolic mixtures. Both testosterone and 7-hydroxycoumarin exhibited a time-dependent decrease.

**Fig. 4:**
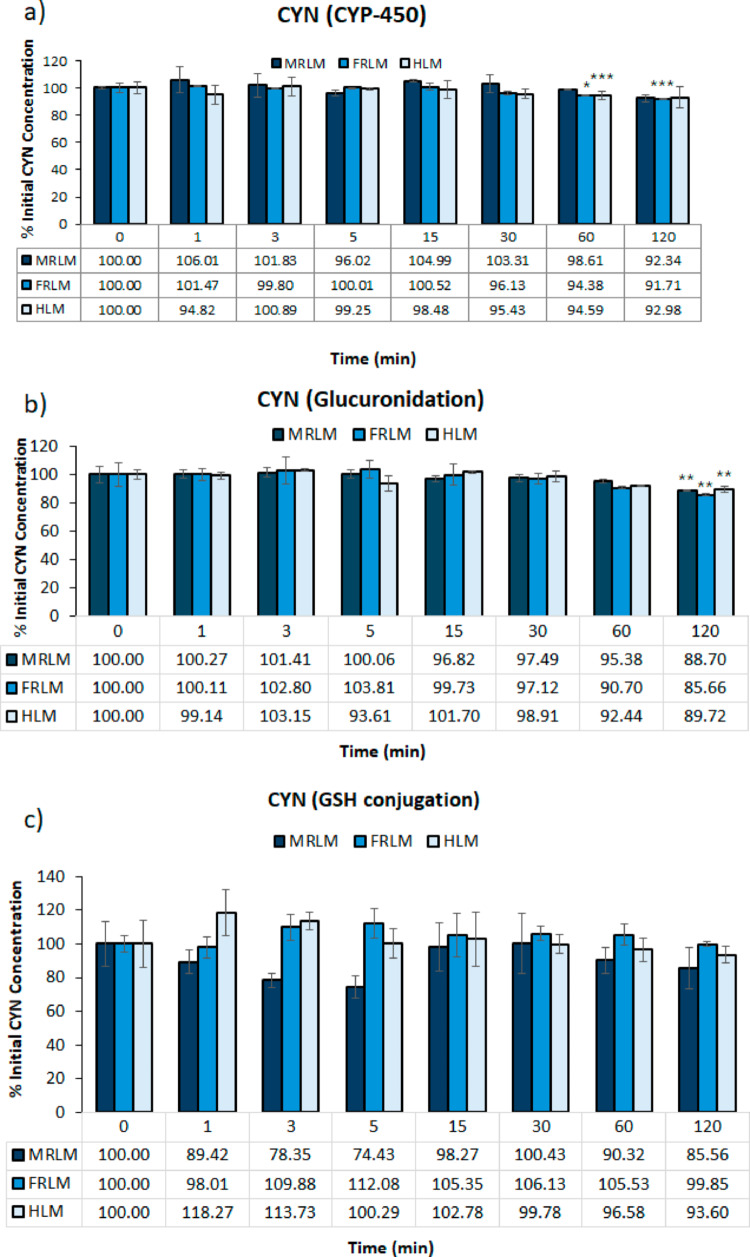
Metabolic stability of CYN incubated with the three different microsomes under the three metabolic reaction conditions: **a** phase I CYP-450; **b** phase II glucuronidation; **c** phase II GSH conjugation. Data are expressed as the means ± SD of triplicate runs

Figure 4 shows the concentration of CYN incubated with the three different microsomes under the three metabolic reaction conditions. Despite the clear metabolic activity demonstrated by the positive controls, CYN exhibited only an ~ 8% decrease after 2 h of incubation in phase I reactions. In contrast, during phase II reactions, a ~ 15% decrease was observed when incubated with glucuronic acid and GSH. While previous studies have demonstrated the high metabolic stability of this toxin (Kittler et al. 2016), the present findings agree with those reported in vivo in the brains of rats exposed to CYN by Plata-Calzado et al. (2025) where it was found that CYN is more susceptible to phase II than to phase I reactions, most likely due to its high hydrophilicity. Indeed, in the present work, most metabolites detected result from phase II reactions. The metabolic activation of CYN via phase I enzymes appears to play only a minor role in its metabolic transformation. Nevertheless, minor metabolism (up to 9%) cannot be entirely ruled out due to methodological limitations (Kittler et al. 2016).

In the present work, the proportion of CYN that was metabolized was reflected in the low concentrations of metabolites identified for the first time in Table 1 (M10–M27). Most of these metabolites resulted from reduction reactions, while only three (M10, M15, and M20) were formed through oxidation reactions. Due to the absence of analytical reference standards, only qualitative characterization was feasible.

**Table 1:** Identification of potential phase I metabolites following in vitro CYN metabolism with male (MRLM) and female (FRLM) rat, and human (HLM) microsomes by UHPLC-MS/MS

AVB	ID	Transformations	Composition Change	m/z error (ppm)	m/z (obs.)	m/z (calc.)
C_15_ H_18_ N_4_ O_3_	M10	Nitro Reduction, Oxidative Deamination to Ketone	− (H_3_ N O_4_ S)	0.12	302.13793	152.07622
C_15_ H_23_ N_5_	M11	Nitro Reduction, Nitro Reduction	− (O_7_ S) + (H_2_)	− 4.97	273.19399	274.20126
C_15_ H_19_ N_5_ O	M12	Dehydration, Nitro Reduction	− (H_2_ O_6_ S)	− 3.83	285.15787	286.16515
C_15_ H_19_ N_5_ O_2_	M13	Desaturation, Nitro Reduction	− (H_2_ O_5_ S)	− 4.46	301.15253	302.15981
C_15_ H_21_ N_5_ O_2_	M14	Nitro Reduction	− (O_5_ S)	− 4.07	303.16829	304.17557
C_15_ H_20_ N_4_ O_3_	M15	Nitro Reduction, Oxidative Deamination to Alcohol	− (H N O_4_ S)	1.93	304.15413	305.16140
C_15_ H_23_ N_5_ O_2_	M16	Nitro Reduction, Reduction	− (O_5_ S) + (H_2_)	− 3.93	305.18398	306.19125
C_15_ H_23_ N_5_ O_3_	M17	Hydration, Nitro Reduction	− (O_4_ S) + (H_2_)	− 4.41	321.17867	322.18595
C_15_ H_21_ N_5_ O_4_	M18	Reduction	− (O_3_ S)	− 1.14	335.15897	336.16625
C_15_ H_17_ N_5_ O_3_	M19	Dehydration	− (H_4_ O_4_ S)	2.93	315.13406	338.12329
C_15_ H_23_ N_5_ O_5_	M20	Double Reduction, Oxidation	− (O_2_ S) + (H_2_)	− 0.81	353.16963	354.17691
C_15_ H_23_ N_5_ O_7_	M21	Dehydration, Double Reduction, Thiourea to Urea	− (S) + (H_2_)	− 3.97	385.15822	386.16550
	M22	Nitro Reduction, Thiourea to Urea	− (S) + (H_2_)	− 3.97	385.15822	386.16550
C_15_ H_25_ N_5_ O_7_	M23	Hydration, Nitro Reduction, Thiourea to Urea	− (S) + (H_4_)	− 4.23	387.17376	388.18104
	M24	Nitro Reduction, Thiourea to Urea	− (S) + (H_4_)	− 4.23	387.17376	388.18104
	M25	Hydration, Nitro Reduction, Thiourea to Urea	− (S) + (H_4_)	− 3.28	387.17413	388.18140
	M26	Nitro Reduction, Thiourea to Urea	− (S) + (H_4_)	− 3.28	387.17413	388.18140
C_15_ H_29_ N_5_ O_5_ S	M27	Double Reduction, Nitro Reduction, Reduction	− (O_2_) + (H_8_)	− 1.09	391.18851	414.17773

Formula	RT (min)	Area (Max.)	MRLM (♂)	FRLM (♀)	HLM
C_15_ H_18_ N_4_ O_3_	1.222	3,983,671	−	−	+
C_15_ H_23_ N_5_	4.809	2,428,771	+	−	+
C_15_ H_19_ N_5_ O	3.203	8,155,305	+	−	+
C_15_ H_19_ N_5_ O_2_	0.725	1,850,030	+	−	−
C_15_ H_21_ N_5_ O_2_	4.549	10,357,984	−	−	+
C_15_ H_20_ N_4_ O_3_	3.779	7,310,508	+	−	−
C_15_ H_23_ N_5_ O_2_	5.016	10,899,284	−	−	+
C_15_ H_23_ N_5_ O_3_	6.175	5,131,763	+	−	−
C_15_ H_21_ N_5_ O_4_	0.687	2,059,485	−	+	−
C_15_ H_17_ N_5_ O_3_	9.187	2,358,116	−	+	−
C_15_ H_23_ N_5_ O_5_	0.688	1,273,790	−	+	−
C_15_ H_23_ N_5_ O_7_	0.618	9,888,641	+	−	−
	0.618	9,888,641	+	−	−
C_15_ H_25_ N_5_ O_7_	0.6	94,014,490	+	−	−
	0.6	94,014,490	+	−	−
	0.808	3,686,547	−	−	+
	0.808	3,686,547	−	−	+
C_15_ H_29_ N_5_ O_5_ S	0.556	2,434,762	−	−	+

Regarding phase II metabolism, the metabolites identified for the first time are listed in Table 2. The vast majority resulted from amino acid conjugation, with cysteine conjugates being particularly prominent, likely derived from GSH conjugation. Additional conjugates with arginine, glycine, taurine, and ornitine were also detected. Furthermore, conjugation with fatty acids such as palmitoyl and stearoyl was observed. Other reactions, including methylation, acetylation, and sulfation, were also identified but appear to represent less frequently utilized metabolic pathways. In addition, several glucuronic acid conjugated metabolites (M70, M72, and M73) have also been identified. The present in vitro results confirm the importance of glycine and glucuronide conjugation reactions already demonstrated in vivo by Plata-Calzado et al. (2025) in the metabolism of CYN, although the metabolites detected in the present study are structurally different from those identified by these authors. Some of these reactions, such as conjugation with glycine, glucuronic acid, or sulfation, had already been predicted by the in silico model. However, it should be noted that the masses obtained from in vitro biotransformation differed, as a preceding phase I reaction was required prior to conjugation, which had not been anticipated by the in silico model.

**Table 2:** Identification of potential phase II metabolites following in vitro CYN metabolism with male (MRLM) and female (FRLM) rat, and human (HLM) microsomes by UHPLC-MS/MS

Formula	ID	Transformations	Composition Change	m/z error (ppm)	m/z (obs.)
C17 H22 N6 O6	M28	Oxidation, Glycine Conjugation	− (O S) + (C2 H N)	− 4.47	406.15827
C16 H25 N5 O6 S	M29	Dehydration, Double Reduction, Methylation	− (O) + (C H4)	0.09	415.15259
	M30	Nitro Reduction, Methylation	− (O) + (C H4)	0.09	415.15259
C20 H27 N7 O6 S	M31	Dehydration, Dehydration, Ornitine Conjugation	− (O) + (C5 H6 N2)	4.7	493.17667
C17 H24 N6 O10 S	M32	Desaturation, Thiourea to Urea, Taurine Conjugation	+ (C2 H3 N O3)	− 2.45	504.12622
	M33	Oxidation, Glycine Conjugation	+ (C2 H3 N O3)	− 2.45	504.12622
C20 H30 N6 O10	M34	Oxidative Deamination to Alcohol, Thiourea to Urea, Ornitine Conjugation	− (S) + (C5 H9 N O3)	4.68	514.20475
C18 H29 N5 O10 S2	M35	Double Reduction, Oxidative Deamination to Ketone, Cysteine Conjugation	+ (C3 H8 O3 S)	0.92	539.13608
C21 H28 N8 O8 S	M36	Dehydration, Oxidative Deamination to Ketone, Arginine Conjugation	+ (C6 H7 N3 O)	3.21	552.17685
C20 H30 N6 O11 S	M37	Hydration, Oxidative Deamination to Alcohol, Glutamine Conjugation	+ (C5 H9 N O4)	0.69	562.16971
	M38	Oxidative Deamination to Alcohol, Glutamine Conjugation	+ (C5 H9 N O4)	0.69	562.16971
C21 H35 N9 O10	M39	Hydration, Thiourea to Urea, Arginine Conjugation	− (S) + (C6 H14 N4 O3)	0.35	573.25089
	M40	Thiourea to Urea, Arginine Conjugation	− (S) + (C6 H14 N4 O3)	0.35	573.25089
C17 H20 N4 O6	M41	Oxidative Deamination to Alcohol, Acetylation	− (H N O S) + (C2)	0.51	376.13848
C17 H28 N6 O4 S	M42	Nitro Reduction, Nitro Reduction, Glycine Conjugation	− (O3) + (C2 H7 N)	− 2.01	412.18845
C20 H29 N7 O4	M43	Nitro Reduction, Glutamine Conjugation	− (O3 S) + (C5 H8 N2)	4.81	431.23018
C17 H19 N5 O7 S	M44	Dehydration, Desaturation, Acetylation	− (H2) + (C2)	− 4.64	437.09849
C17 H27 N5 O9	M45	Double Reduction, Thiourea to Urea, Acetylation	− (S) + (C2 H6 O2)	− 3.7	445.17923
C17 H21 N5 O10	M46	Oxidative Deamination to Ketone, Thiourea to Urea, Glycine Conjugation	− (S) + (C2 O3)	3.22	455.13031
C15 H20 N4 O12 S	M47	Oxidative Deamination to Alcohol, Thiourea to Urea, Sulfation	− (H N) + (O5)	4.78	480.08214
C18 H28 N6 O8 S	M48	Nitro Reduction, Thiourea to Urea, Cysteine Conjugation	+ (C3 H7 N O)	4.44	488.1711
C17 H27 N5 O9 S	M49	Double Reduction, Oxidative Deamination to Alcohol, Glycine Conjugation	+ (C2 H6 O2)	− 1.89	477.15204
C17 H27 N5 O9 S	M50	Double Reduction, Acetylation	+ (C2 H6 O2)	− 1.89	477.15204
C17 H27 N5 O9 S	M51	Reduction, Acetylation	+ (C2 H6 O2)	− 1.89	477.15204
C17 H23 N5 O9 S2	M52	Dehydration, Oxidative Deamination to Alcohol, Taurine Conjugation	+ (C2 H2 O2 S)	− 4.27	505.09156
C15 H25 N5 O11 S	M53	Double Reduction, Thiourea to Urea, Sulfation	+ (H4 O4)	1.11	483.12766
C17 H23 N5 O11 S	M54	Oxidative Deamination to Ketone, Thiourea to Urea, Taurine Conjugation	+ (C2 H2 O4)	− 3.7	505.10961
C17 H28 N6 O8 S2	M55	Dehydration, Double Reduction, Taurine Conjugation	+ (C2 H7 N O S)	− 1.86	508.14006
	M56	Nitro Reduction, Taurine Conjugation	+ (C2 H7 N O S)	− 1.86	508.14006
C17 H25 N5 O8 S2	M57	Nitro Reduction, Oxidative Deamination to Ketone, Taurine Conjugation	+ (C2 H4 O S)	− 4.18	491.1124
C18 H24 N6 O10 S	M58	Desaturation, Thiourea to Urea, Cysteine Conjugation	+ (C3 H3 N O3)	3.96	516.1295
C18 H30 N6 O8 S2	M59	Dehydration, Double Reduction, Cysteine Conjugation	+ (C3 H9 N O S)	− 4.45	522.15433
	M60	Nitro Reduction, Cysteine Conjugation	+ (C3 H9 N O S)	− 4.45	522.15433
C17 H30 N6 O9 S2	M61	Double Reduction, Taurine Conjugation	+ (C2 H9 N O2 S)	− 1.66	526.15069
C18 H28 N6 O8 S2	M62	Dehydration, Reduction, Cysteine Conjugation	+ (C3 H7 N O S)	1.46	520.14176
C18 H28 N6 O8 S2	M63	Nitro Reduction, Cysteine Conjugation	+ (C3 H7 N O S)	1.46	520.14176
C21 H39 N9 O6 S	M64	Double Reduction, Nitro Reduction, Arginine Conjugation	− (O) + (C6 H18 N4)	1.6	545.27527
C21 H35 N9 O10	M65	Hydration, Thiourea to Urea, Arginine Conjugation	− (S) + (C6 H14 N4 O3)	− 4.49	573.24811
	M66	Thiourea to Urea, Arginine Conjugation	− (S) + (C6 H14 N4 O3)	− 4.49	573.24811
C21 H32 N8 O10	M67	Oxidative Deamination to Alcohol, Thiourea to Urea, Arginine Conjugation	− (S) + (C6 H11 N3 O3)	− 4.58	556.22159
C31 H51 N5 O6	M68	Hydration, Palmitoyl Conjugation	− (O S) + (C16 H30)	− 2.71	589.38234
C33 H51 N5 O4	M69	Dehydration, Stearyl Conjugation	− (O3 S) + (C18 H30)	1.64	581.39506
C21 H24 N4 O14 S	M70	Desaturation, Oxidative Deamination to Ketone, Glucuronide Conjugation	− (N) + (C6 H3 O7)	0.01	588.10098
C33 H52 N4 O6	M71	Oxidative Deamination to Alcohol, Stearyl Conjugation	− (N O S) + (C18 H31)	− 5.04	600.38566
C21 H26 N4 O15 S	M72	Oxidation, Oxidative Deamination to Ketone, Glucuronide Conjugation	− (N) + (C6 H5 O8)	− 0.05	606.11151
	M73	Oxidative Deamination to Ketone, Glucuronide Conjugation	− (N) + (C6 H5 O8)	− 0.05	606.11151
C33 H53 N5 O8	M74	Dehydration, Thiourea to Urea, Stearyl Conjugation	− (S) + (C18 H32 O)	− 3.71	647.38701
C33 H57 N5 O6 S	M75	Nitro Reduction, Stearyl Conjugation	− (O) + (C18 H36)	2.4	651.40452
C33 H53 N5 O9	M76	Desaturation, Thiourea to Urea, Stearyl Conjugation	− (S) + (C18 H32 O2)	2.68	663.38611
C25 H42 N8 O9 S2	M77	Nitro Reduction, Nitro Reduction, GSH Conjugation	+ (C10 H21 N3 O2 S)	− 3.22	662.24949
C33 H59 N5 O9	M78	Double Reduction, Thiourea to Urea, Stearyl Conjugation	− (S) + (C18 H38 O2)	− 5.15	669.42783

Formula	m/z (calc.)	RT (min)	Area (Max.)	MRLM (♂)	FRLM (♀)	HLM
C17 H22 N6 O6	204.08641	0.634	1,577,551	−	−	+
C16 H25 N5 O6 S	208.58357	0.855	1,301,017	−	−	+
	208.58357	0.855	1,301,017	−	−	+
C20 H27 N7 O6 S	247.59561	7.758	1,782,309	−	+	−
C17 H24 N6 O10 S	253.07039	0.602	4,796,722	−	−	+
	253.07039	0.602	4,796,722	−	−	+
C20 H30 N6 O10	258.10965	0.585	566,965	−	−	+
C18 H29 N5 O10 S2	270.57532	1.928	3,840,266	−	−	+
C21 H28 N8 O8 S	277.09570	7.756	1,486,921	−	+	−
C20 H30 N6 O11 S	282.09213	0.678	680,866	+	−	−
	282.09213	0.678	680,866	+	−	−
C21 H35 N9 O10	287.63272	8.186	1,573,350	−	+	−
	287.63272	8.186	1,573,350	−	+	−
C17 H20 N4 O6	377.14575	4.335	2,898,619	+	−	−
C17 H28 N6 O4 S	413.19572	7.844	2,792,414	+	−	−
C20 H29 N7 O4	432.23745	7.637	25,316,468	−	+	+
C17 H19 N5 O7 S	438.10577	1.97	26,569,751	+	−	+
C17 H27 N5 O9	446.18651	0.601	24,815,606	+	−	−
C17 H21 N5 O10	456.13758	0.722	6,610,526	+	−	−
C15 H20 N4 O12 S	481.08941	0.674	3,506,714	+	−	−
C18 H28 N6 O8 S	489.17838	6.196	1,060,132	−	−	+
C17 H27 N5 O9 S	500.14127	0.74	56,366,205	−	−	+
C17 H27 N5 O9 S	500.14127	0.74	56,366,205	−	−	+
C17 H27 N5 O9 S	500.14127	0.74	56,366,205	−	−	+
C17 H23 N5 O9 S2	506.09884	0.674	1,006,758	+	−	−
C15 H25 N5 O11 S	506.11688	0.721	2,009,432	+	−	−
C17 H23 N5 O11 S	506.11688	0.721	1,984,796	+	−	−
C17 H28 N6 O8 S2	509.14733	0.616	13,864,407	+	−	−
	509.14733	0.616	13,864,407	+	−	−
C17 H25 N5 O8 S2	514.10162	0.706	926,909	−	−	+
C18 H24 N6 O10 S	517.13678	7.756	1,200,475	−	+	−
C18 H30 N6 O8 S2	523.16160	0.607	6,449,148	−	−	+
	523.16160	0.607	6,449,148	−	−	+
C17 H30 N6 O9 S2	527.15797	0.627	49,505,769	+	−	−
C18 H28 N6 O8 S2	543.13098	0.606	2,252,434	−	+	−
C18 H28 N6 O8 S2	543.13098	0.606	2,252,434	−	+	−
C21 H39 N9 O6 S	546.28255	8.445	8,363,794	+	−	−
C21 H35 N9 O10	574.25539	6.933	5,524,016	+	−	−
	574.25539	6.933	5,524,016	+	−	−
C21 H32 N8 O10	579.21081	6.933	9,028,439	+	+	−
C31 H51 N5 O6	590.38961	8.624	5,623,033	+	−	−
C33 H51 N5 O4	604.38428	8.665	2,444,324	−	−	+
C21 H24 N4 O14 S	611.09020	0.703	1,184,401	+	−	−
C33 H52 N4 O6	623.37488	8.768	1,677,676	+	−	−
C21 H26 N4 O15 S	629.10073	2.165	5,014,206	−	−	+
	629.10073	2.165	5,014,206	−	−	+
C33 H53 N5 O8	648.39429	7.842	5,606,779	−	+	−
C33 H57 N5 O6 S	652.41179	4.889	3,267,013	+	+	−
C33 H53 N5 O9	664.39338	8.949	7,490,936	−	+	−
C25 H42 N8 O9 S2	685.23871	0.626	4,841,219	+	−	−
C33 H59 N5 O9	692.41705	9.183	877,349	−	+	−

The results indicate that there are no significant quantitative metabolic differences between species or between sexes, as the proportion of metabolized CYN was comparable across all microsomes tested (male rat, female rat, and human) in the three different metabolic reactions. This suggests that the overall metabolic stability of CYN is consistent regardless of the biological source. Nevertheless, when examining the specific metabolites generated, some variability was observed. Only a subset of metabolites (specifically M11, M12, M43, M67, and M75) was consistently detected across multiple microsomes, while the other appeared to be species- or sex specific. This finding highlights that, although no quantitative differences were observed, there is a wide range of metabolic possibilities at qualitative level.

CYN is recognized as a relatively persistent cyanotoxin in freshwater systems, with reported resistance to degradation under environmentally relevant conditions (Kinnear 2010; Scarlett et al. 2020). The limited biotransformation observed in our study (8–15%) supports the notion that CYN may remain bioavailable after uptake, potentially contributing to prolonged internal exposure. In addition, several studies have reported the accumulation of CYN in aquatic organisms such as fish, as well as in edible plants irrigated with contaminated water (de la Cruz et al. 2013; Duque et al. 2025), highlighting its relevance for trophic transfer and human exposure. Although evidence for biomagnification remains limited, the combination of environmental persistence and metabolic stability underscores the importance of considering CYN within long-term exposure.

In the present study, the predominance of amino acid conjugates among phase II metabolites suggests that CYN undergoes biotransformation pathways consistent with general detoxification mechanisms (Hodges and Minich 2015). Nevertheless, the requirement of prior phase I reduction indicates that intermediate metabolites may be generated before conjugation, and these intermediates could potentially exhibit different reactivity or biological activity (Li et al. 2011). As described in the literature, an imbalance between phase I activation and phase II conjugation may lead to the accumulation of reactive intermediates capable of interacting with cellular macromolecules, thereby contributing to toxicity (Potęga 2022).

The divergence between computational and experimental data highlights a well-recognized limitation of current in silico approaches, which are largely based on rule-based systems and training datasets that may not fully capture enzyme specificity, sequential metabolic pathways, or the complexity of biotransformation in biological systems. Furthermore, these tools typically operate at the level of individual compounds and do not account for mixture effects, enzyme competition, or cofactor availability, all of which can influence metabolic outcomes (Kar and Leszczynski 2019).

Further studies are needed to address the different routes of cyanotoxins biotransformation and the implication of metabolites in their toxicity. In addition, it is necessary to address the role of microbial metabolism in the fate of CYN which is likely to play an important role in its fate under natural conditions, as has been described for other cyanotoxins. For instance, in the case of MCs, microbial degradation pathways have been identified, including ring cleavage by bacteria such as *Sphingopyxis* sp., revealing the existence of complementary non-enzymatic detoxification mechanisms (Ding et al. 2018). In fact, a decrease in CYN concentration has also been demonstrated following exposure to colonic bacteria, further suggesting the relevance of microbial metabolism in its detoxification (Diez-Quijada et al., 2020). Upcoming studies should address in vivo validation of these pathways, the role of microbial metabolism, and the implications of co-exposure with other contaminants for human and environmental health.

## Conclusions

This study provides for the first time the integrative assessment of CYN metabolism through a combined in silico ADMET approach and in vitro assays using human and rat liver microsomes. Computational modeling predicted limited absorption, distribution, and CYP450-mediated metabolism of CYN, while highlighting potential conjugation routes and the formation of metabolites with variable toxicological profiles. Complementary in vitro assays confirmed the high metabolic stability of CYN, with only modest decreases in toxin levels observed after incubation (8–15%). Particularly, glucuronidation and amino acid conjugation (phase II reactions), including cysteine, glycine, taurine, and arginine played a more prominent role than phase I oxidation, resulting in the identification of multiple novel metabolites. No major quantitative differences were detected between sexes or species, although qualitative variability in the metabolites generated suggests multiple possible biotransformation routes. Overall, this work advances the understanding of CYN toxicokinetics by providing the first evidence of its conjugated metabolites in vitro and supports the use of integrative in silico and in vitro approaches for risk assessment.
